# Reappraisal of anoxic spreading depolarization as a terminal event during oxygen–glucose deprivation in brain slices in vitro

**DOI:** 10.1038/s41598-020-75975-w

**Published:** 2020-11-04

**Authors:** Elvira Juzekaeva, Azat Gainutdinov, Marat Mukhtarov, Roustem Khazipov

**Affiliations:** 1 Laboratory of Neurobiology, Kazan Federal University, Kazan, 420008 Russia; 2 INMED/INSERM UMR1249, Aix-Marseille University, 163 Avenue de Luminy BP13, 13273 Marseille Cedex 09, France

**Keywords:** Neuroscience, Diseases of the nervous system, Stroke

## Abstract

Anoxic spreading depolarization (aSD) has been hypothesized as a terminal event during oxygen–glucose deprivation (OGD) in submerged cortical slices in vitro. However, mechanical artifacts caused by aSD-triggered edema may introduce error in the assessment of neuronal viability. Here, using continuous patch-clamp recordings from submerged rat cortical slices, we first confirmed that vast majority of L4 neurons permanently lost their membrane potential during OGD-induced aSD. In some recordings, spontaneous transition from whole-cell to out-side out configuration occurred during or after aSD, and only a small fraction of neurons survived aSD with reperfusion started shortly after aSD. Secondly, to minimize artifacts caused by OGD-induced edema, cells were short-term patched following OGD episodes of various duration. Nearly half of L4 cells maintained membrane potential and showed the ability to spike-fire if reperfusion started less than 10 min after aSD. The probability of finding live neurons progressively decreased at longer reperfusion delays at a rate of about 2% per minute. We also found that neurons in L2/3 show nearly threefold higher resistance to OGD than neurons in L4. Our results suggest that in the OGD ischemia model, aSD is not a terminal event, and that the “commitment point” of irreversible damage occurs at variable delays, in the range of tens of minutes, after OGD-induced aSD in submerged cortical slices.

## Introduction

Duration of ischemia is the key factor determining neuronal survival, neurological, and behavioral outcome. During global brain ischemia caused by cardiac arrest, irreversible loss of brain function and legal death are conventionally thought to occur within 5–10 min of cardiac arrest^1^. Paradoxically, however, neuronal function can be restored even after much longer periods of global ischemia under several experimental settings including extracorporeal reperfusion with special cytoprotective reperfusate up to 4 h after cardiac arrest in pigs^2^ or maintenance of rat brain slices that were prepared 1–6 h after cardiac arrest^3^, or after 30 min middle cerebral artery occlusion^4^, in ACSF. These observations raised the hypothesis that neurons may survive much longer episodes of ischemia than traditionally thought, thus opening questions for further research on the possibility of expanding the time window for resuscitation.

Development of brain injury during ischemia proceeds through the initial phase of compensation, during which brain activity ceases but neurons maintain their membrane potential, and recovery of cardiovascular function restores brain activity without major damage to the brain^1,5,6^. Passage to the decompensation phase is associated with a wave of collective and nearly complete neuronal depolarization, so-called anoxic spreading depolarization (aSD) occurring at ~ 5 min after the onset of ischemia^5,7,8^. Generation of aSD is caused by depletion of intracellular reserves of energy metabolites, loss of function of the energy-dependent active ion transporters and rupture in transmembrane ionic gradients^5,7,8^. aSD is a highly energy-demanding event which severely aggravates metabolic status^9^. Considerable evidence indicates that aSD is the key ischemic event that triggers cascades of intracellular reactions leading to acute and delayed neuronal death^5,7,8^. Similarly, periinfarct depolarizations (PIDs) severely compromise the metabolic state in the penumbra and cause expansion of the ischemic core during the development of the focal ischemic injury^10^.

The oxygen–glucose deprivation (OGD) model of an ischemia-like condition has been extensively used to explore acute changes in neuronal function during metabolic insult in brain slices in vitro. In this model, metabolite deprivation causes a series of events largely recapitulating the development of ischemic injury in vivo, including aSD and ischemic neuronal death. OGD-induced aSD develops within 5–10 min of the onset of perfusion with OGD-solution, it is associated with a wave of collective neuronal depolarization and a drop in electrical membrane resistance, large negative shifts of the extracellular local field potential (LFP) and an increase in tissue light transmittance^11–16^. OGD-induced aSD has been described in slices of neocortex^13,16–20^, hippocampus^12,14,15,21–24^, striatum^25^, thalamus^26,27^ and cerebellar cortex^28^, but not subthalamic brain structures^18,26,29^. Also, aSD heralds the development of tissue edema manifested by the expansion of brain slice borders, elevation of the slice surface, and massive tissue shifts within the slice^30,31^. However, at difference to ischemia in vivo, where neuronal functions can be, at least partially, preserved when perfusion/oxygen supply is reestablished at delay of 30 min–6 h after the ischemia onset^2–5^, occurrence of aSD is considered as a “terminal”, i.e. irreversible event during OGD in submerged slices in vitro^8^. Indeed, intracellular sharp electrode recordings from submerged hippocampal slices revealed that CA1 neurons irreversibly lost their membrane potential and synaptic responses during OGD even if reoxygenation started 1 min after aSD^14,15^. Similarly, whole-cell recordings from neocortex and hippocampus in vitro showed that even when oxygen and glucose were reintroduced to the brain slice immediately or few minutes after aSD, the membrane potential was irreversibly lost^12,16,18,21^. Along with these findings, extracellular recordings of responses evoked by afferents electrical stimulation were permanently lost during OGD even if reperfusion with oxygenated ACSF started shortly after aSD both in hippocampus and neocortex^12,13,20–22^. These observations led to the conclusion that at difference to aSD in vivo, aSD in submerged slices of cortex and hippocampus is a lethal terminal event^12–16,18,20–22^. However, massive tissue shifts and mechanical displacement of neurons from the recording electrodes caused by tissue swelling, which starts to develop after aSD^30–32^ could severely compromise recordings and introduce error in the assessment of neuronal viability. Therefore, in the present study we reexamined the hypothesis of aSD as a terminal event during OGD, taking into account the methodical constraints imposed by ischemic edema. In this aim, we estimated neuronal survival by short-term patching of neurons following reperfusion started at various delays after aSD. We provide evidence that aSD is not a terminal event during OGD in submerged cortical slices, as previously thought, and that the “commitment point” of irreversible loss of membrane potential and neuronal function occurs at variable and much longer delays, in the range of tens of minutes, after aSD, in the OGD model of ischemia in vitro. Our findings also suggest that the OGD model of ischemia in vitro could be useful in further explorations of neuroprotective interventions that could stop the progression of neuronal damage initiated by aSD during the post-aSD period.

## Results

### Continuous patch-clamp recordings through OGD and reperfusion

In the present study we explored neuronal survival during OGD in slices of the barrel cortex using whole-cell patch clamp recordings and concomitant extracellular recordings of LFP, and optical intrinsic signals (OIS) imaging. To this aim we used two types of neuronal survival assessment. In the first approach, we performed continuous recordings from a single neuron starting from control conditions and then through OGD and reperfusion. In the second approach, we sampled cells using short-term patch-clamp recordings in control conditions and during reperfusion following OGD of various durations (from 1 to 60 min). Both approaches were used in the same slices (n = 33) of the rat barrel cortex.

**Figure 1:**
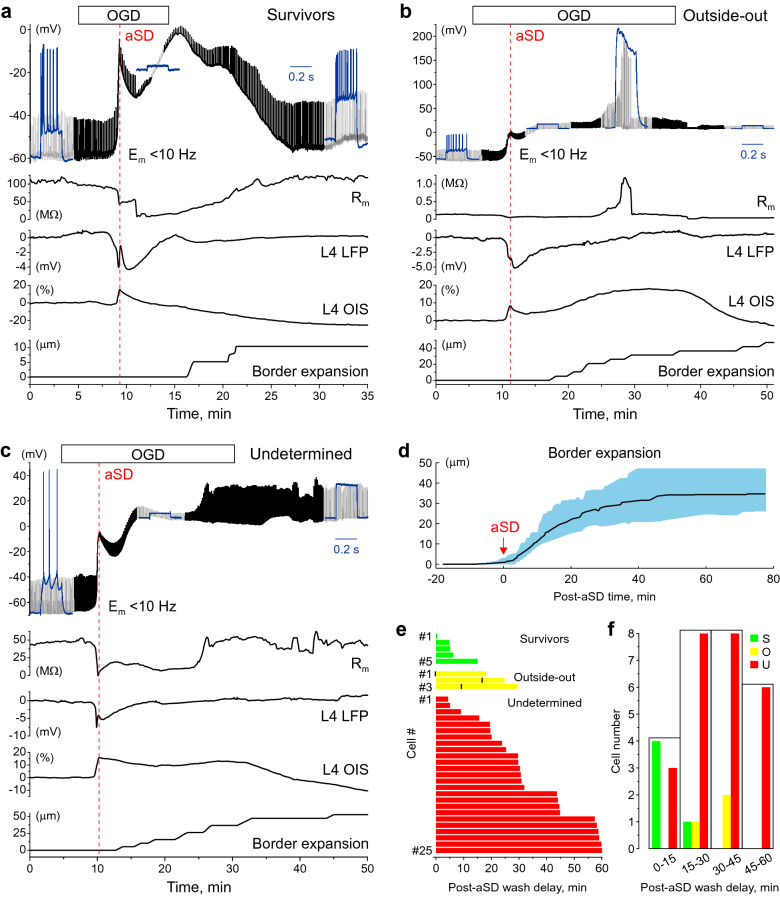
Variety of electrophysiological outcomes during continuous whole-cell recordings through the oxygen–glucose deprivation and reperfusion. **(a–c)** Example traces of continuous recordings from L4 neurons in current-clamp mode starting from control conditions and then through OGD of various durations and then reperfusion. The upper black traces show E_m_ (10 Hz low-pass filtered to remove spikes), regular upward deflections show E_m_ deflections evoked by depolarizing steps to monitor cell firing and R_m_. Blue insets show cell responses (non-filtered signal) to depolarizing current steps in control conditions, after OGD-induced aSD and after reperfusion. Below, the corresponding changes in membrane resistance, DC-LFP and OIS recordings from L4, and the time course of the OGD-induced border expansion from control position are shown. Example traces for three categories of cells depending on their functional outcome after OGD: (**a**) Survivor, which transiently depolarized during aSD but recovered E_m_, R_m_ and firing ability during reperfusion; (**b**) Cell lost during transition to outside-out, manifested as a sharp increase in R_m_ during reperfusion to > 1GΩ values and (**c**) Undetermined case which showed no recovery of E_m_ nor ability for spike firing upon reperfusion. (**d**) Average change in the OGD-induced border expansion (shaded area shows SD). Data are referenced to aSD time = 0. (**e**) Raster plot of outcomes (as shown on panels **a**–**c**) as a function of reperfusion delay after OGD-induced aSD. For outside-out transitions, the time points of transition to “gigaseal” are indicated by black vertical bars. (**f**) Histograms of the cell count in each outcome category depending on reperfusion delay after aSD. (**d**,**e**) Group data from 33 L4 neurons.

**Figure 2:**
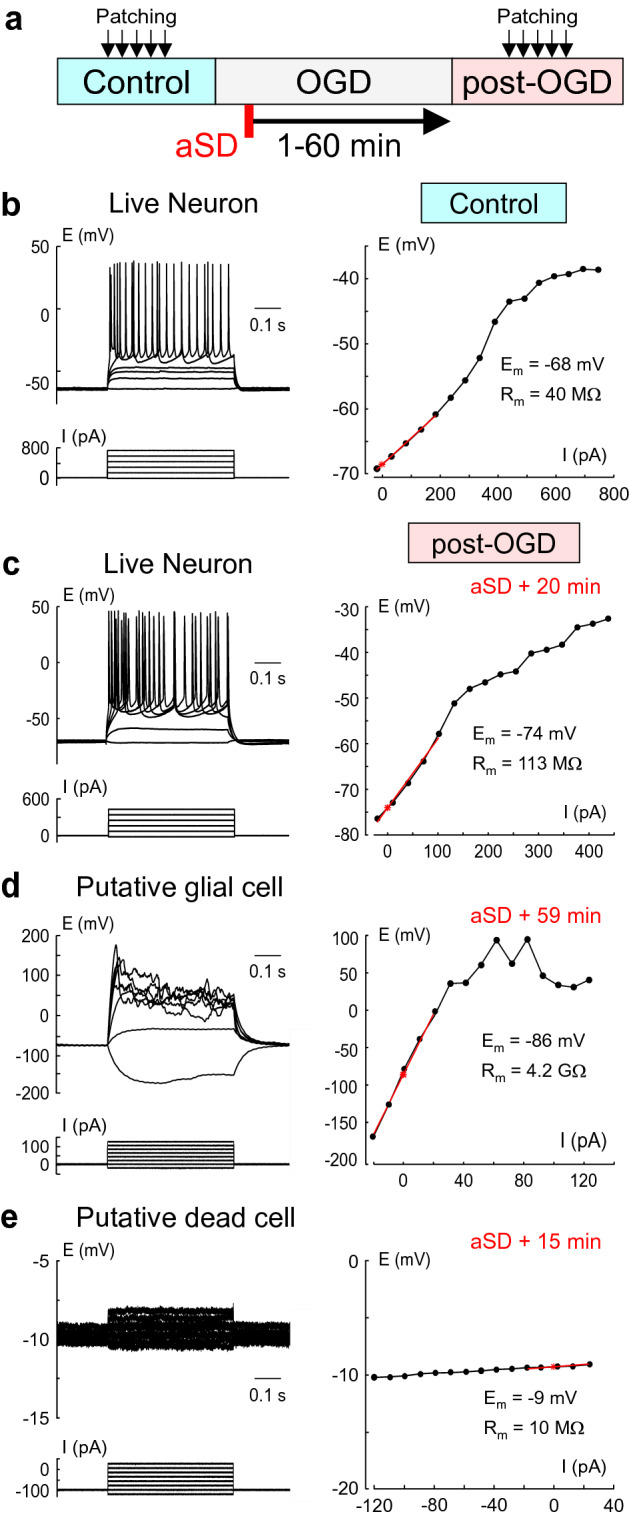
Variety of electrophysiological outcomes from OGD using short-term whole-cell recordings. (**a**) Scheme of the experiment. Cells were sampled using short-term (~ 1 min long) patch-clamp recordings in control conditions and during reperfusion following OGD of various durations (from 1 to 60 min after aSD). (**b**) Voltage responses (top traces) generated by current injections (bottom traces) recorded from a L4 neuron prior to OGD. The corresponding I-V plot is shown on the right, where the red line shows the range in which R_m_ was measured, and E_m_ and R_m_ are indicated on the right of the I-V curve. (**c**) Example traces obtained from a L4 neuron which survived OGD with reperfusion started 20 min after aSD. (**d**) L4 cell which survived OGD with reperfusion started 59 min after aSD, and which has been classified as a putative glial cell due to very negative E_m_, high R_m_ and inability for spike firing. (**e**) Putative dead cell with low R_m_ and strongly depolarized E_m_.

During continuous recordings, under control conditions L4 neurons had a membrane potential (E_m_) of (median (Q1–Q3): − 68.4 (− 74.8 to − 61.7) mV, a membrane resistance (R_m_) of 96.3 (55.7–132.1) MΩ and fired APs in response to suprathreshold depolarizing current steps, which were applied every 10 s to monitor the change in R_m_ through the entire duration of recordings (Fig. 1a–c, note that E_m_ traces are low-pass filtered to remove spikes, and that E_m_ corresponds to the bottom values of the E_m_ trace). Perfusion with OGD-solution evoked aSD in all neurons at a delay of 5.1 (4.3–8.2) min after OGD onset. aSD was manifested by rapid neuronal depolarization to − 13.6 (− 27.9 to − 7.2) mV (p = 3.0E−12, hereinafter Wilcoxon rank sum test) and occurred simultaneously with a negative shift in extracellular LFP of − 5.6 (− 8.7 to − 3.1) mV in concomitant DC-LFP recordings in the vicinity of the recorded neuron, and an increase in light transparency by 15.3 (9.4–21.3) % in OIS recordings. During aSD, R_m_ dropped to 10.3 (4.1–29.7) MΩ (p = 6.7E−08). Also, ischemic edema and mechanical slice tissue displacement started developing shortly after aSD as assessed by expansion of the slice borders (Fig. 1d). These observations are in keeping with previous studies using the OGD model^13,16–19,22,31–34^. Further developments after aSD varied between cells and depended on OGD duration (Fig. 1e,f). We sorted neurons into three categories depending on the functional outcome after OGD, based on the parameters E_m_, R_m_ and ability to fire APs in response to depolarizing steps: (1) survivors (Fig. 1a); (2) cells lost during recordings due to a transition from whole-cell to outside-out (Fig. 1b); and (3) cells which apparently did not show functional recovery after OGD, but due to uncertainty in the cause (cell death or cell loss due to a loss of electrical contact with the cell due to displacement during tissue edema) were classified as undetermined cases (Fig. 1c).

#### Survivors

Only a small minority of L4 neurons recovered during reperfusion (n = 5 neurons of 33, 15%) when reperfusion was started shortly, within a few minutes, after aSD (5.2 (3.7–8.9) min) (Fig. 1a,e,f). Only one neuron survived an OGD episode in which reperfusion started 15 min after aSD. Survivor neurons displayed an E_m_ of − 70.6 (− 76.1 to − 47.2) mV and R_m_ of 119.9 (111.5–156.6) MΩ compared to control values for this group of − 75.3 (− 77.9 to − 61.4) mV (p = 0.55) and 100.9 (83.9–132.9) MΩ (p = 0.31), and they recovered their ability to fire APs upon repolarization during reperfusion. Used as a metric of edema, border expansion had a tendency to start later 6.7 (2.4–9.2) min, and attain lower values of 21.2 (18.4–39.6) μm compared to other outcomes: 2.3 (− 1.1 to 4.4) min and 40.6 (29.9–55.0) μm for outside-out cases and 3.7 (1.5–5.4) min and 36.6 (30.2–44.6) μm for undetermined cases, but this difference was not significant (p = 0.29 for Survivors/Outside-out, p = 0.29 for Survivors/Undetermined and p = 0.67 for Outside-out/Undetermined).

#### Lost during transition to outside-out

Three cells of 33 (9%) showed spontaneous transition from whole-cell to outside-out configuration as evidenced by an increase in R_m_ to > 1 GΩ (1.9 (1.3–2.0) GΩ, range from 1.1 to 2.1 GΩ) (Fig. 1b,e,f). This transition occurred at variable delays after aSD (9.2 (1.9–14.8) min) during OGD episodes in which reperfusion started from 17.6 to 29.3 min (24.8 (19.4–28.1) min) after aSD, and it was typically transient with further rupture of the patch evidenced by a drop in R_m_. Neither E_m_ [− 1.3 (− 5.5 to 6.6) mV] compared to control value of − 57.5 (− 63.7 to − 56.2) mV (p = 0.029) nor the ability to fire APs recovered in these cases.

#### Undetermined

This was the most frequent category of outcomes from OGD (n = 25 cells of 33, 76%) observed through the entire span of OGD durations (reperfusion delay after aSD from 4 to 60 min, average 30.7 (23.6–48.6) min) (Fig. 1c,e,f). All undetermined cases did not show recovery of E_m_ during reperfusion (− 2.3 (− 8.2 to 6.3) mV), measured 10–15 min after reperfusion onset, compared to control value of − 69.2 (− 74.4 to − 61.9) mV (p = 1.4E−09) and displayed a variable change in R_m_ (from 3.4 to 723.1 MΩ), average 54.6 (28.4–208.3) MΩ, compared to control value of 89.0 (54.2–116.6) MΩ (p = 0.07). None of these cases showed APs in response to depolarizing steps from negative, close to pre-OGD E_m_ maintained by constant negative current injection (not shown). These predominant cases of outcomes were classified as dead neurons in previous studies^12,14–16,18^. However, taking into account the major methodical constraints imposed by ischemic edema^31–33^ which starts to develop after aSD, and which may severely compromise recordings (as clearly evidenced by transitions to outside-out) we assigned this category as undetermined cases with the hypothesis that some of these cells actually survive but are lost during recordings due to edemic tissue displacement and thus the survival rate is probably underestimated during continuous recordings.

### Short-term patch-clamp recordings

**Figure 3:**
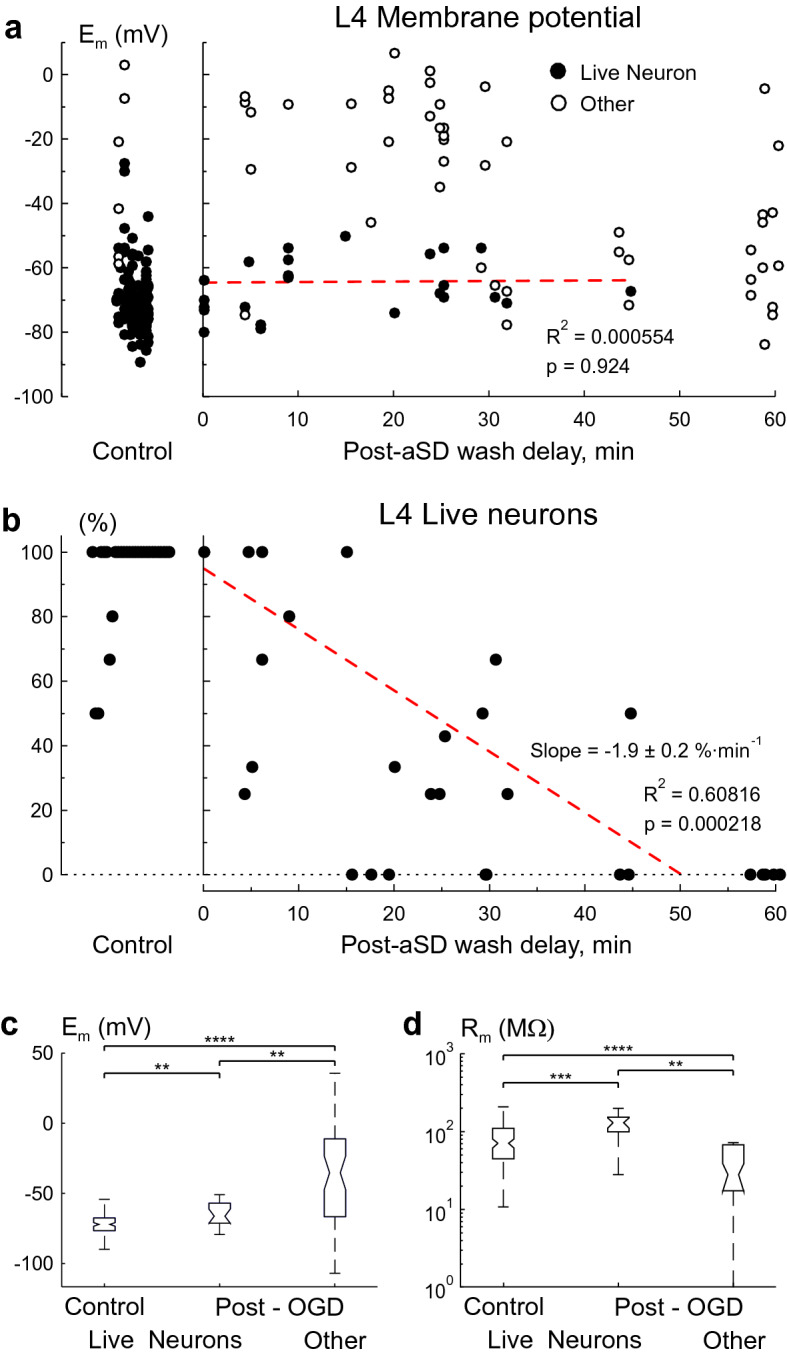
OGD survival in L4 neurons using short-term patching approach. (**a**) E_m_ of cells patched in L4 before (left) and after OGD episodes (right) as a function of the onset of reperfusion delay after aSD. Each data point corresponds to an individual cell. Filled circles represent live L4 neurons with negative membrane potentials and the ability to fire spontaneously or in response to depolarizing steps. Open circles show data from “other” types of cells which did not fire action potentials as the main criterion for their exclusion from a pool of live neurons. The red dotted line shows no dependence of E_m_ in survivor neurons on the onset of reperfusion delay after aSD. Data from 128 control and 69 post-OGD cells. (**b**) The corresponding proportion of live neurons among all cells patched in L4 in a given slice (each dot represents one slice). The red dotted line represents a linear fit for the probability of finding a live neuron in L4 as a function of the onset of reperfusion delay after aSD. The intercept was fixed to the control value of cells viability of 95%. Data from 29 slices. (**c**) E_m_ and (**d**) R_m_ values in L4 in live neurons before OGD, and live L4 neurons and “other” cells in the post-OGD epoch (boxplots show median and 1st and 3nd quarantiles). Group data from 128 control and 69 post-OGD cells in 29 slices. (**p < 0.01, ***p < 0.001, ****p < 0.0001).

To overcome the problem of edema we short-term patched cells during the reperfusion phase after OGD of various durations. Recording sessions started 20 min after reperfusion onset and lasted in each cell for ~ 1 min. According to the estimates of slice surface displacement during OGD-induced edema (~ 2 μm min^−1^^31^), cell displacement from the patch-pipette during such short-term recordings should not affect assessment of neuronal viability. For each cell patched in these experiments we estimated E_m_, R_m_ and ability to fire APs in response to depolarizing current steps before and after OGD episodes (Fig. 2a). Under control conditions, 122 of 128 (95%) cells from 29 slices patched in L4 displayed features of live neurons with a membrane potential (E_m_) of − 71.5 (− 75.9 to − 66.9) mV, membrane resistance (R_m_) of 104.8 (73.5–148.8) MΩ, and APs in response to suprathreshold depolarizing current steps (Figs. 2b, 3a,c,d). The remaining 6 (5%) cells had a depolarized E_m_ of − 31.1 (− 56.6 to − 7.3) mV, R_m_ of 240.0 (38.0–380.7) MΩ, and did not fire in response to depolarizing steps from − 60/− 70 mV. Cells patched after reperfusion in the post-OGD epoch were classified according to similar criteria as for the continuous recordings above. These included two main categories, (1) Live neurons–survivors, and (2) Others.

1. Live neurons in post-OGD slices (n = 19, 28% of the total 69 patched cells from 29 slices) were characterized by an E_m_ of − 65.6 (− 70.6 to − 56.4) mV, that was + 5.9 mV more positive than that in control conditions (p = 0.0056)^35,36^, R_m_ of 145.7 (129.9–176.6) MΩ, that was 1.5 fold higher than that in control conditions (p = 0.0012), and they all fired APs in response to depolarizing steps from rest (Figs. 2c, 3a,c,d). Neither E_m_ (R^2^ = 0.000554, p = 0.924) nor R_m_ (R^2^ = 0.045, p = 0.383) showed any correlation with the time delay of reperfusion after aSD. Live neurons could be found in OGD-exposed slices with reperfusion started at delay of up to 45 min after aSD (Fig. 3a,b). The proportion of live neurons among all cells patched in a slice was highly variable between slices and progressively decreased with increase in the delay of reperfusion onset after aSD (Fig. 3b). The linear fit provided a slope of − 1.9 ± 0.2%·min^−1^ for the probability of finding live cells in L4 as a function of delay of reperfusion after aSD.
2. The other L4 cells patched in post-OGD slices (n = 50, 72% of the total 69 patched cells from 29 slices) displayed high variability in electrophysiological characteristics and included live putative glial cells with very negative E_m_ values, some of them presumably of oligodendrocyte lineage displaying high input resistance and active electrogenic properties (Fig. 2d), and presumably dead cells of unknown origin with strongly depolarized or nearly zero membrane potential and extremely low membrane resistance (Fig. 2e). On average, these other cells in post-OGD slices had an E_m_ of − 34.9 (− 65.9 to − 10.9) mV (p = 1E−10) and R_m_ of 41.2 (28.9–68.6) MΩ (p = 4.3E−12) (Fig. 3c,d). None of the “other” type cells fired APs in response to depolarizing current steps.

**Figure 4:**
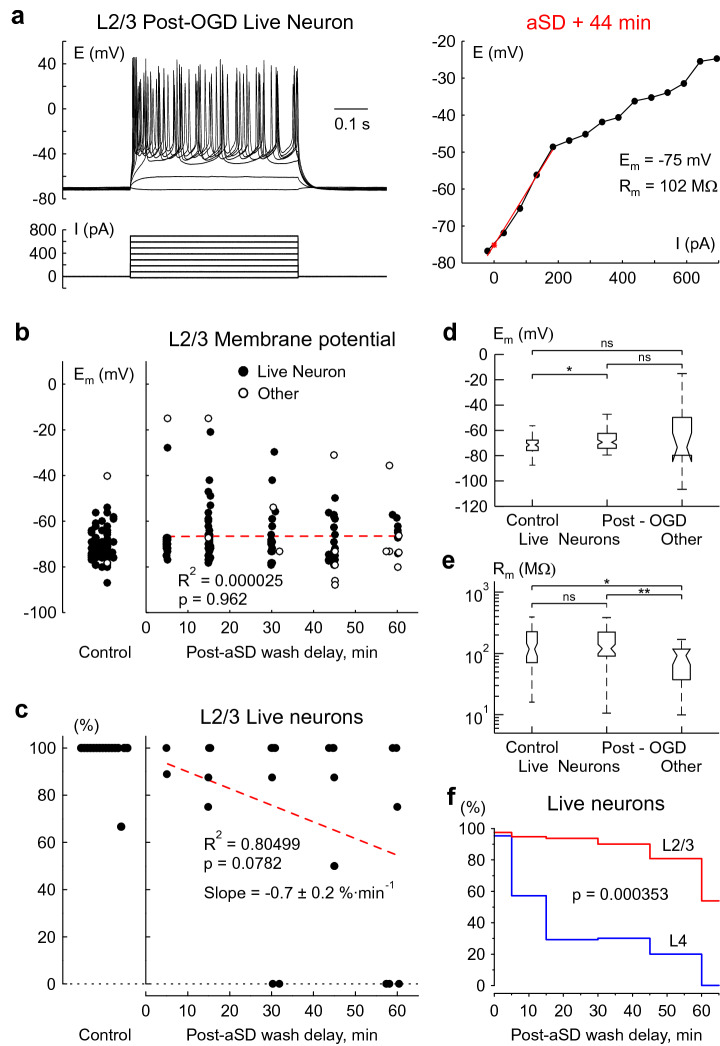
Neurons in L2/3 show higher resistance to OGD than L4 neurons. (**a**) Voltage responses generated by current injections in a L2/3 neuron in a slice which experienced OGD for more than 1 h, and in which reperfusion started at a delay of 59 min after aSD. Right, the corresponding I-V plot. (**b**) E_m_ values in cells patched in L2/3 before (left) and after OGD of various reperfusion delays after aSD onset (right). Each data point corresponds to an individual cell. Filled circles correspond to live L2/3 neurons, open circles correspond to “other” cells. The red dotted line shows no dependence of E_m_ in survivor neurons on the onset of reperfusion delay after aSD. Data from 79 control and 122 post-OGD cells. (**c**) The corresponding proportion of live neurons among all cells patched in L2/3 in a given slice (each dot represents one slice). The red dotted line represents a linear fit for the probability of finding a live neuron in L2/3 as a function of the onset of reperfusion delay after aSD. The intercept was fixed to the control value of cells viability of 97%. Data from 16 control and 25 post-OGD slices. (**d**) E_m_ and (**e**) R_m_ values in L2/3 in live neurons before OGD, and live L2/3 neurons and “other” cells in the post-OGD epoch (boxplots show median and 1st and 3nd quarantiles). Group data from 79 control cells in 16 slices and 122 post-OGD cells in 25 slices. (*p < 0.05, **p < 0.01). (**f**) Histogram plots of the probability of finding a live neuron in L2/3 and L4 as a function of the onset of reperfusion delay after aSD. Group data from 122 L2/3 cells in 25 slices and 69 L4 cells in 29 slices. P-value is for a comparison between L2/3 and L4 using Kolmogorov–Smirnov test.

We also sampled cells in L2/3 using a similar approach as for L4. In control conditions 97% of cells (n = 77 of the total 79 patched cells from 16 slices) appeared to be live neurons with an ability to fire action potentials in response to depolarizing steps, E_m_ of − 71.1 (− 75.5 to − 67.3) mV and R_m_ of 119.5 (72.2–224.7) MΩ (Fig. 4b,d,e). In slices exposed to OGD and with reperfusion started 5–60 min after aSD, live neurons accounted the vast majority of all cells patched in L2/3 (n = 100, 82% of the total 122 patched cells from 25 slices; Fig. 4a,b) had an E_m_ of − 69.0 (− 73.8 to − 62.0) mV depolarized by 2.1 mV comparing to the control value (p = 0.0124), and R_m_ of 121.8 (91.7–224.8) MΩ that was not different from the control (p = 0.38) (Fig. 4d,e). As in L4, neither E_m_ (R^2^ = 0.000025, p = 0.962) nor R_m_ (R^2^ = 0.000909, p = 0.769) of the OGD-surviving neurons showed any correlation with the time delay of reperfusion after aSD (Fig. 4b). With an increase in the OGD duration, the proportion of surviving neurons decreased by a slope of − 0.7 ± 0.2%·min^−1^ that was about threefold slower than for L4 (Fig. 4c). Even in slices that were reperfused at a delay of 60 min after aSD 54% of patched cells were live neurons (n = 7 of the total 13 patched cells) (Fig. 4b,f). The “other” L2/3 cells patched in post-OGD slices (n = 22, 18% of the total 122 patched cells from 25 slices) fired no APs in response to depolarizing current steps. On average, these “other” cells had an E_m_ of − 73.0 (− 79.3 to − 49.5) mV (p = 0.9921) and R_m_ of 93.78 (37.9–119.6) MΩ (p = 0.0104) (Fig. 4d,e). Analysis of probability of finding live neurons through the post-OGD period revealed that L2/3 neurons showed a much higher survival rate after aSD when compared to L4 neurons (p = 0.000353, Kolmogorov–Smirnov test, Fig. 4f).

## Discussion

The main conclusion of our study is that aSD is not a terminal event during OGD in submerged cortical slices, and that the “commitment point” of irreversible damage in this model occurs at variable, in the range of tens of minutes, delays after aSD.

Here we used two experimental approaches to assess neuronal survival after transient metabolic insult caused by OGD. Using continuous patch-clamp recordings through the OGD and reperfusion period we found an extremely low survival rate with recovery of negative membrane potential and ability to spike-fire in very few neurons and only if reperfusion started shortly after aSD. This is in line with previous studies where the occurrence of aSD was associated with irreversible loss of membrane potential in submerged slices or intact preparations^12–16,18,21^. However, according to recent observations on the dynamics of tissue edema which starts developing after aSD at a rate of volume increase by ~ 1%/min, irreversible loss of membrane potential after aSD could reflect not only true permanent neuronal depolarization, but also artifacts caused by mechanical displacement of recorded neurons from the recording pipette, and loss of electrical connection between the cell and pipette. This could potentially cause cell survival to be underestimated. In line with these assumptions, we found spontaneous transitions from whole-cell to outside-out configuration following aSD. Normally, transition from whole-cell to outside-out configuration involves slow displacement of the recording pipette from a cell, during which the initial connection between the pipette and cell membrane is preserved and once a relatively small patch is extruded from the plasma membrane, the outside-out formation manifests in an increase in R_m_ to GΩ values^37^. Although infrequent, cases of transitions from whole-cell to outside-out configuration are highly likely to be indicative of the displacement of the recorded cell from the pipette during edemic tissue shifts. Transition to outside-out is not necessarily indicative of cell damage, however, as neurons can survive this procedure and display quite normal negative E_m_ values during multiple repatching under normal conditions. Therefore, these cases can only be classified as a fraction of undetermined outcomes, that in total constitute the vast majority of cases during continuous recordings through the OGD and reperfusion phases. To sum up, continuous recordings employed in this, and previous studies show poor neuronal survival after aSD, but this approach may underestimate survival rate due to the artifacts caused by ischemic tissue edema. Likewise irreversible loss of responses evoked by afferents electrical stimulation during extracellular recordings that has been reported after aSD in brain slices^12,13,20–22^ could also involve slice tissue displacement relatively the stimulating and recording electrodes as well as mechanical neuronal trauma caused by the brain mass tissue movement against the electrodes during development of ischemic edema.

The short-term patching approach, in which the mechanical artifacts associated with tissue edema were minimized to displacement of only a few microns over ~ 1 min of recording time, showed neuronal death that was more protracted after aSD. Indeed, nearly half of L4 neurons survived OGD episodes during which reperfusion started less than 10 min after aSD, and survival probability decayed with a time constant of 21 min as a function of reperfusion delay after aSD. An even higher survival rate has been found in the supragranular layers, where a half of patched cells were live neurons in slices in which reperfusion was delayed from aSD by 60 min. The higher susceptibility of L4 neurons to OGD is consistent with a “tropism” of aSD to L4 and the higher metabolic demand of this layer as evidenced by the heaviest labelling of L4 by the metabolic level marker 2,3,5-triphenyltetrazolium chloride (TTC)^20^, and is in keeping with heterogeneity in sensitivities to ischemia among different neuronal populations and structures^7,11,18,26,29,38–40^ that may involve different metabolism in different cell types, but also different neuronal microenvironment which plays a key role in neuronal recovery from OGD^41,42^. Because continuous recordings through the OGD and reperfusion period contain a major source of error caused by tissue edema, we propose that the short-term patching approach, in which this error is minimized, provides more reliable estimates of neuronal survival. However, several points should be taken into account during the interpretation of these results. First, the proportion of surviving neurons could be overestimated with the short-term patching approach because dead cells may change in appearance and ability to establish gigaseal contact and thus can be overlooked during patching. Second, surviving neurons showing functional recovery of membrane potential and firing ability upon reperfusion may have already initiated the cascade of intracellular processes which leads to neuronal death later. Third, cells that were classified as “others” or “undetermined” cases, which show a lack of firing, loss of E_m_ and low membrane resistance within 1–2 h of reperfusion may regain their function at later time points. Therefore, our conclusions on neuronal survival after OGD are limited to the present experimental settings and may be different with other approaches and at other time points after OGD.

Our findings on significant survival of neurons and their protracted death after aSD reappraise the role of aSD as a terminal event in the OGD model in submerged slices or intact preparations in vitro^12–16,18,20–22,43^ and are more consistent with the observations made using other ischemia models. Indeed, in brain slices with a gas-fluid interface, aSD is not a terminal event and slices can be rescued by reperfusion with oxygen/glucose-containing ACSF several minutes after hypoxia-induced aSD (in normal glycemic conditions)^8,44,45^. During global ischemia in vivo, aSD is also not a terminal event as both morphological and functional impairments to cortical neurons are fully reversible upon reperfusion started within ~ 5 min of aSD^6^. Moreover, cortical slices prepared from the rat brain 1–6 h after cardiac arrest display quite normal physiological and morphological properties^3^ and sufficient viability for cultures^46–48^. Specific cellular functions, spontaneous synaptic activity, and active cerebral metabolism (yet not global brain activity) was restored in pigs four hours post-mortem after extracorporeal reperfusion using cytoprotective perfusate^2^. Also, reperfusion started after one hour of complete global ischemia could result in neuronal, electrophysiological, and metabolic brain resuscitation^49–52^. Also, along with these findings, up to 4 h of reperfusion after a transient (one hour long) focal cerebral ischemia induced by microfilament MCA occlusion, negligible tissue injury was revealed if brain slices prepared from these animals were stained with TTC in vitro^53^. No signs of functional damage were also reported in slices prepared immediately after 30 min of MCAO^4^.

Comparing our results with the data obtained in vivo or using *ex-vivo* preparations, it appears that neuronal death occurs at a higher rate during OGD in submerged slices than during ischemia in vivo. The more harmful OGD in submerged slices may involve a stronger cytotoxic edema and calcium load than in vivo. Previously, the development of cytotoxic edema manifested by swelling of neuronal and glial cell soma, dendritic beading and shrinkage of the extracellular space was shown starting minutes after aSD both in vivo and in vitro^6,22,32,54–56^. However, under submerged conditions in vitro, brain tissue is exposed to an unlimited source of calcium, sodium, chloride and water in the extracellular space, through continuous and unlimited buffering of the extracellular space by the electrolytes and water contained in the bath medium^31,34,57,58^. This results in a stronger cytotoxic edema and calcium load than in vivo, where the availability of electrolytes and water in the extracellular space is limited by the lack of blood supply to the ischemic core in the case of focal ischemia or during global brain ischemia caused by cardiac arrest^59^. In the latter case, cytotoxic edema and calcium load are limited by the relatively small amount of electrolytes and water in the narrow extracellular space and are thus less pronounced and less damaging, and occur without tissue swelling in contrast to submerged slices in vitro^31,34,57,58^.

In conclusion, the results of the present study suggest that neuronal death evoked by OGD in submerged cortical slices may span a longer time window after aSD than initially thought, and this is more in agreement with the observations made during brain ischemia in vivo. While previously approved strategies for attenuating ischemic injury in the in vitro ischemia models are mainly aimed at delaying aSD onset^12,13,22,24,43^, our findings allow in vitro models to be used to explore neuroprotective interventions that could stop the progression of neuronal damage initiated by aSD during the post-aSD period.

## Materials and methods

### Ethical approval

All animal-use protocols followed the guidelines of the French National Institute of Health and Medical Research (INSERM, protocol N007.08.01) and Kazan Federal University on the use of laboratory animals (ethical approval by the Institutional Animal Care and Use Committee of Kazan State Medical University N9-2013).

### Brain slice preparation

Wistar rats (16–23 days old) of either sex were used. Animals were decapitated under isoflurane anaesthesia (5%), the brain was rapidly removed and placed in ice-cold (2–5 °C) slicing solution of the following composition (in mM): KCl 3.5, CaCl_2_ 2, MgSO_4_ 2, NaHCO_3_ 24, NaH_2_PO_4_ 1.25, glucose 10 and sucrose 25.2 (pH 7.4). Four hundred µm thick thalamocortical slices were cut using a PELCO easiSlicer vibratome (Ted Pella, Inc., Redding, CA, USA). Slices containing the barrel cortex were selected by anatomical coordinates^60^ and the presence of barrel structures in L4. Slices were first kept in oxygenated (95% O_2_–5% CO_2_) artificial cerebrospinal fluid (ACSF) of the following composition (in mM): NaCl 126, KCl 3.5, CaCl_2_ 2, MgCl_2_ 1.3, NaHCO_3_ 25, NaH_2_PO_4_ 1.2 and glucose 11 (pH 7.4) for 30 min at 32 °C and then at room temperature (20–22 °C) for at least 1 h before use. For recordings, slices were placed into a submerged chamber and superfused with oxygenated ACSF at 30–32 °C at a flow rate of 10 ml min^−1^. Oxygen/glucose deprivation (OGD) was induced by superfusion with ACSF in which N_2_ replaced O_2_ and sucrose replaced glucose at equimolar concentration. OGD durations were fixed prior to each experiment to a pre-defined duration of the post-aSD period, which is a time interval between aSD (monitored using OIS imaging and extracellular field potential recordings) and reperfusion with oxygenated, enriched in glucose ACSF. Pre-defined durations of the post-aSD period were of 0, 5, 10, 15, 20, 25, 30, 45 and 60 min during recordings from L4, and 5, 15, 30, 45 and 60 min during recordings from L2/3. The actual durations of post-aSD period could differ by up to ± 2 min from the pre-defined values due to the time lag between aSD detected online during the experiment and the exact value of the aSD peak determined during post-hoc analysis.

### Electrophysiological recordings

#### Patch-clamp recordings

Visual patch-clamp recordings were performed from L4 pyramidal cells using a MultiClamp 700B (Axon Instruments, Union City, CA, USA) amplifier as described previously^31^. Patch electrodes were made from borosilicate glass capillaries (BF150-86-10, Sutter Instrument, Novato, CA, USA) and had a resistance of 4–7 MΩ. The pipette (intracellular) solution contained (in mM) 131 potassium gluconate, 4 KCl, 10 HEPES, 10 phosphocreatine, 4 MgATP, and 0.3 Na_2_GTP (adjusted to pH 7.3 with KOH). Extracellular and patch-clamp recordings were digitized at 10 kHz with a Digidata 1440A interface card (Axon Instruments) and analyzed offline using MATLAB (MathWorks, Inc., Natick, MA, USA) routines.

To examine the ability of neurons to fire APs and monitor the membrane resistance (R_m_) during continuous patch-clamp recordings 200-ms suprathreshold depolarizing current pulses were applied every 10 s during control, OGD and reperfusion episodes. For short-term patch-clamp recordings in each slice, several neurons were patched before OGD (on average, 4.4 ± 0.4 cells patched per slice in L4 (128 cells in 29 slices) and 4.9 ± 0.4 cells/slice in L2/3 (79 cells in 16 slices) and after OGD (on average, 2.4 ± 0.3 cells/slice in L4 (69 cells in 29 slices) and 4.9 ± 0.7 cells/slice in L2/3 (122 cells in 25 slices). Firing ability, resting membrane potential (E_m_) and R_m_ values in these cells were evaluated by I-V curve analysis.

Extracellular recordings of the local field potentials (LFP) were performed in the barrel cortex using single site glass pipette electrodes. Electrodes were pulled from borosilicate glass capillaries (BF150-86-10, Sutter Instrument, Novato, CA, USA) and had resistances of 2–3 MΩ when filled with ACSF. Electrodes were connected via chlorided silver wire to the headstage of a MultiClamp 700B patch-clamp amplifier (Axon Instruments, Union City, CA, USA). Recordings were performed in voltage-clamp DC mode, then currents were inverted and voltage calibrated using 5 mV steps.

#### Optical intrinsic signal recordings

Optical intrinsic signal (OIS) recordings were performed using slice transillumination as described in^20,31,61^. The slice was illuminated by a halogen lamp with a 775 nm bandpass filter and visualized using a BX51WI upright microscope equipped with a 4x/0.10 Plan N objective (Olympus, Tokyo, Japan). Images were acquired using a QIClick-R-F-M-12 CCD camera (QImaging, Surrey, BC, Canada) every 2.5 s at 348 × 260 pixel resolution.

### Data analysis

Data were analyzed using custom-written procedures in MATLAB (MathWorks, Inc., Natick, MA, USA) as described previously^20,31^. OIS was calculated using the first-frame subtraction approach: OIS(t) = (I(t) − I_0_)/I_0_, where I(t)—pixel intensity at the moment t, I_0_—time-averaged pixel intensity in the preconditioned baseline period (100 s). The resulting frames were filtered with a 10 × 10 median filter. Regions of interest (ROI) were selected as square areas near recording sites. OIS traces were calculated as the average OIS signal within the selected ROIs. The appearance of the peak value of the OIS signal determined the start time of aSD. The amplitude of aSD during extracellular recordings was calculated as within OIS-detected aSD ± 5 min time window.

### Statistical analysis

Statistical analysis was performed using the MATLAB Statistics toolbox (MathWorks, Inc., Natick, MA, USA). Statistical comparisons between the groups were performed using the Wilcoxon rank sum and Kolmogorov–Smirnov tests. The significance level was set at p < 0.05. Group data are expressed as median (25th percentile–75th percentile) unless otherwise indicated.

## Data Availability

Original and processed data, and signal processing and analysis routines are available on request from the authors.
